# Supplementary material for: HIV-1 integrase tetramers are the antiviral target of pyridine-based allosteric integrase inhibitors
Source: eLife. 2019 May 23;8:e46344. doi: 10.7554/eLife.46344 (PMC6581505; doi:10.7554/eLife.46344)
Supplement: Supplementary file 1. [file elife-46344-supp1.docx]

**Supplementary File 1:**

**Chemistry-General methods:**

Unless stated otherwise, reactions were monitored by thin layer chromatography (TLC) on silica gel plates (60 F254), visualizing with ultraviolet light or *p*-anisaldehyde. Dichloromethane (CH_2_Cl_2_), tetrahydrofuran (THF), diethyl ether, toluene, and dimethylformamide (DMF) were purified with a Pure Solv. MD-6 solvent purification system. All other solvents and reagents were used as received. Column chromatography was performed on silica gel (60–120 mesh) using hexanes and ethyl acetate. Solvents for chromatography are listed as a percentage (v/v). ^1^H and ^13^C NMR spectra were recorded on a Bruker DPX 400 spectrometer in CDCl_3_ or DMSO-*d_6_* solutions operating at 400 MHz for ^1^H NMR and 100 MHz for ^13^C NMR. Chemical shifts were reported in ppm on the δ scale relative to residual CHCl_3_ (δ = 7.28 for ^1^H NMR and δ = 77.2 for ^13^C NMR) as an internal reference. Spin multiplicities are given as s (singlet), d (doublet), t (triplet), and m (multiplet) as well as b (broad). Coupling constants (*J*) are given in hertz (Hz). MS spectra were obtained on a mass spectrometer. ESI mass spectra were measured on an Thermo LTQ Orbitrap instrument. Optical rotations were measured on an Anton Paar MCP 150 polarimeter.

**Synthetic route to enantiopure KF116**


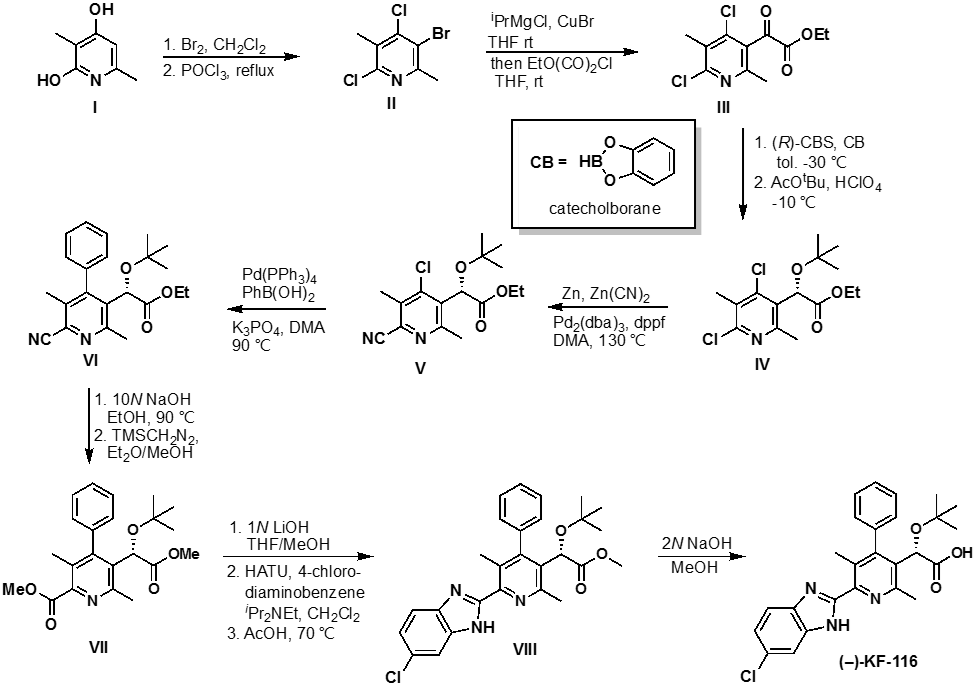


**3-bromo-4,6-dichloro-2,5-dimethylpyridine II**

Bromine (1.5 mL, 29.32), pre-dissolved in CH_2_Cl_2_ (30 mL), was added dropwise to dihydroxypyridine **I** (4 g, 28.76mmol) suspended in CH_2_Cl_2_ (30 mL) at room temperature. The resulting mixture was stirred for 1 h and the resulting precipitated solid was filtered to give 5-bromo-3,6-dimethylpyridine-2,4-diol as the HBr salt. The dried HBr salt was heated to reflux in POCl_3_ (30 mL) for 17 h and cooled to room temperature. The excess POCl_3_ was distilled off under vacuum and the residue was poured onto ice. The pH of the mixture was made basic (pH > 10) using 10 N NaOH. The mixture was filtered and the filtrate was extracted with CH_2_Cl_2_ (4 x 50 mL) and dried over sodium sulfate. Flash chromatography (SiO_2_, 5% ethyl acetate in hexanes) afforded brominated pyridine **II.** ^1^H NMR (CDCl_3_) δ 2.65 (s, 3H), 2.50 (s, 3H). ^13^CNMR δ (CDCl_3_) δ 156.2, 149.3, 145.8, 129.7, 120.8, 25.8, 18.2.

**Ethyl 2-(4,6-dichloro-2,5-dimethylpyridin-3-yl)-2-oxoacetate III**

*i*-PrMgCl (2.31 mL, 2.M M in THF, 4.62 mmol) was added dropwise to a mixture of dichlorobromopyridine **II** (590 mg, 2.31 mmol) and CuBr (83 mg, 0.5786 mmol) in anhydrous THF (88 mL) under argon was at rt. After 1 h, neat ethyl oxalyl chloride (0.54 mL, 4.86 mmol) was added dropwise and allowed to stir for 1 h at rt. After completion of the reaction as monitored by TLC, the reaction was quenched with saturated aqueous NaHCO_3_ solution and extracted with EtOAc (3 x 5 mL). The combined organic extracts was washed with water and brine and dried over Na_2_SO_4_. After removal of the solvents in vacuo the crude material was purified by silica gel column chromatography to provide **III** (461 mg, 72 %) as a light yellow oil (solidifies upon sitting in the freezer. ^1^H NMR (400 MHz, CDCl_3_) δ 4.40 (q, *J* = 7.1 2H), 2.46 (s, 3H), 2.45 (s, 3H), 1.39 (t, *J* = 7.1 Hz, 3H).

# Procedure for the synthesis of both enantiomers of ethyl (*S*))-2-(*tert*-butoxy)-2-(4,6-dichloro-2,5-dimethylpyridin-3-yl)a cetate IV

# A mixture of ethyl 2-(4,6-dichloro-2,5-dimethylpyridin-3-yl)-2-oxoacetate III (270 mg, 0.978 mmol) and (*R*)-(+)-2-Methyl-CBS-oxazaborolidine (0.20 mL, 0.20 mmol, 1.0 M in toluene) in anhydrous toluene (10 mL) was placed in a 50-mL round bottom flask. The mixture was cooled to -20 ºC. To this cold mixture was added a solution of catecholborane (CB) in THF (1.46 mL, 1.46 mmol, 1.0 M). The mixture was stirred at -20 ºC for 2 hours (monitored by TLC) and diluted with EtOAc (50 mL) followed by addition of 15% aqueous solution of Na_2_CO_3_ (10 mL). The mixture was stirred vigorously for 15 min and the layers were then separated. The procedure was repeated (2x) with 10 mL of 15% aqueous Na_2_CO_3_. The organic layer obtained was washed with water, brine and dried over Na_2_SO_4_. After filtration, the solvent was removed and the crude residue purified by silica gel chromatography using 10, 20 and 25% EtOAc/Hex to afford desired ethyl (*S*)-2-(4,6-dichloro-2,5-dimethylpyridin-3-yl)-2-hydroxyacetate (230 mg, 85% yield) contaminated with some catechol borane. The (*R*)-enantiomer was prepared using this same procedure by employing the antipode of the catalyst (*S*)-(+)-2-Methyl-CBS-oxazaborolidine. A sample was purified for analysis by flash column chromatography. *ee* > 95 was determined by Mosher ester analysis.

# A solution of ethyl (*S*)-2-(4,6-dichloro-2,5-dimethylpyridin-3-yl)-2-hydroxyacetate 4 (250 mg, 0.90 mmol) in *tert*-butyl acetate (18 mL, 0.05 M) was cooled in an ice-salt bath. To the sealed, cooled solution was added 70% perchloric acid (0.78 mL, 9.00 mmol). The mixture was stirred for 3 h at -5 °C and monitored by TLC. Upon completion of the reaction, the mixture was diluted with EtOAc (20 mL) and carefully quenched with saturated solution of NaHCO_3_. The mixture was transferred to a separatory funnel and the layers were separated. The organic layer was washed twice with saturated NaHCO_3_, water, brine, dried over Na_2_SO_4_ and concentrated. Silica gel chromatography (Si_2_O, 10 % EtOAc in hexanes) of the crude provided title compound IV (230 mg, 77 %) as a colorless oil. ^1^H NMR (400 MHz, CDCl_3_) δ 5.69 (s, 1H), 4.16 (q, *J* = 7.1 Hz, 2H), 2.56 (d, *J* = 0.5 Hz, 3H), 2.47 (d, *J* = 0.5 Hz, 3H), 1.20 (s, 9H) 1.19 (t, *J* = 7.1, 3H). ^13^C NMR (100 MHz, CDCl_3_) δ 170.0, 159.0, 144.2, 135.6, 134.1, 132.3, 115.7, 76.4, 69.5, 61.5, 27.6, 27.5, 23.2, 17.2, 13.8.

**Procedure for the synthesis of both enantiomers of Ethyl 2-(*tert*-butoxy)-2-(4-chloro-6-cyano-2,5-dimethylpyridin-3-yl)acetate V**

Dichloropyridine **IV** (250 mg, 0.74 mmol), Pd_2_(dba)_3_ (34 mg, 0.037 mmol), dppf (42 mg, 0.075 mmol), Zn powder (6.5 mg, 0.075 mmol), Zn(CN)_2_ (88 mg, 0.75 mmol), were taken in degassed anhydrous DMA (2.0 mL) and heated in a sealed tube at 130 °C overnight. The mixture was then cooled to room temperature, quenched with water and extracted with EtOAc (3 x 5 mL). The combined organic layers were washed with brine, dried over Na_2_SO_4_ and concentrated. Silica gel chromatography (Si_2_O, 13 % EtOAc in hexanes) provided **V (**222 mg, 91%) as a yellow oil. ^1^H NMR (400 MHz, CDCl_3_) δ 5.75 (s, 1H), 4.18 (q, *J* = 7.1 Hz, 2H), 2.62 (s, 3H), 2.60 (s, 3H), 1.2 (t, *J* = 7.1, 3H). ^13^C NMR (100 MHz, CDCl_3_) δ 170.6, 159.7, 144.8, 136.1, 134.7, 132.9, 116.3, 77.1, 70.1, 62.1, 28.2, 23.7, 17.8, 14.4.

**Procedure for the synthesis of both enantiomers of** **Ethyl 2-(*tert*-butoxy)-2-(6-cyano-2,5-dimethyl-4-phenylpyridin-3-yl)acetate VI**

A solution of chloropyridine **V** (135 mg, 0.37 mmol), phenylboronic acid (101 mg, 0.83 mmol), tetrakis(triphenylphosphine)palladium(0) (214 mg, 0.19 mmol), K_3_PO_4_ (0.69 mL, 1.39 mmol, 2.0 M aqueous solution) in 1,4-dioxane (4.6 mL) was degassed by bubbling argon through the solution for 5 min. The mixture was then heated at 110 °C for 16 h in a sealed tube. The reaction mixture was cooled to room temperature and diluted with EtOAc, washed with water followed by brine. The organic layer was dried over sodium sulfate, filtered and concentrated. Flash chromatography (10% EtOAc in hexanes) provided coupling product **VI** (111 mg, 82%) as thick clear oil. ^1^H NMR (400 MHz, CDCl_3_) δ 7.53–7.43 (m, 3H), 7.18-7.14 (m, 2H), 4.91 (s, 1H), 4.18 (q, *J* = 7.1 Hz, 2H), 3.65 (s, 3H), 2.20 (s, 3H), 1.24 (t, 3H), 0.95 (s, 9H).

**Procedure for the synthesis of both enantiomers of** **Methyl 5-(1-(*tert*-butoxy)-2-methoxy-2-oxoethyl)-3,6-dimethyl-4-phenylpicolinate VII**

To a solution of picolinonitrile **VI** (111 mg, 0.303 mmol) in EtOH (0.61 mL) was added 10*N* aqueous NaOH solution (0.30 mL). The mixture was heated at 90 °C for 16 h in a sealed tube. The reaction mixture was then cooled to room temperature and neutralized with 2*N* aqueous HCl solution. The mixture was extracted with EtOAc (3 x 5 mL) and the combined organic layers were dried over Na_2_SO_4_, and concentrated to provide the crude diacid (65 mg, 76 %). This was taken further to next step without further purification.

# The crude material was dissolved in (1:10) MeOH:Ether (3.0 mL), treated dropwise with a 2M solution of (trimethylsilyl)diazomethane in hexanes (0.76 mL, 1.52 mmol), and then stirred at room temperature for 10 min. The reaction mixture was then concentrated to dryness in vacuo. Flash chromatography (20% EtOAc in hexanes) provided dimethyl ester VII as colorless oil (85 mg, 73% 2 steps). ^1^H NMR (400 MHz, CDCl_3_) δ 7.49 – 7.40 (m, 3H), 7.27 – 7.21 (m, 1H), 7.16 – 7.10 (m, 1H), 4.93 (s, 1H), 3.98 (d, *J* = 6.0 Hz, 3H), 3.68 (s, 3H), 2.65 (s, 3H), 2.10 (s, 3H), 0.95 (s, 9H). ^13^C NMR (100 MHz, CDCl_3_) δ 172.4, 167.5, 156.0, 150.7, 147.271, 136.9, 133.7, 129.4, 128.9, 128.6, 128.6, 128.3, 128.2, 76.0, 70.3, 52.7, 52.3, 27.9, 23.5, 16.8.

# Procedure for the synthesis of both enantiomers of Methyl 2-(*tert*-butoxy)-2-(6-(6-chloro-1H-benzo[d]imidazol-2-yl)-2,5-dimethyl-4-phenylpyridin-3-yl)acetate VIII

1*N* LiOH (0.29 mL, 0.31 mmol) was added to dimethyl ester **VII** (85 mg, 0.22 mmol) in THF (2.1 mL), MeOH (0.9 mL) and the mixture was stirred overnight (~16 h) at room temperature. The reaction mixture was then acidified with 1 *N* HCl and diluted with brine and extracted with EtOAc (3 x 5 mL). The combined organic layers were washed with brine, dried over Na_2_SO_4_, filtered and concentrated to give the crude acid which was used the next step without further purification.

Diisopropylethylamine (0.184 mL, 1.056 mmol) was added to a solution of crude acid, 4-chloro-*o*-phenylenediamine (112 mg, 0.0.44 mmol), and HATU (168 mg, 0.44 mmol) dissolved in DMF (2.2 mL). The mixture was stirred overnight at room temperature under argon. The reaction mixture was then diluted with EtOAc and water and the aqueous layer was extracted with EtOAc (3 x 5 mL). The combined organic layers were washed with brine, dried over sodium sulfate, filtered, and concentrated.

The crude amide was then taken up in glacial acetic acid (2 mL) and heated to 70 °C for 40 min. The solution was then concentrated under reduced pressure, diluted with EtOAc and washed with saturated aqueous NaHCO_3_ and brine. The organic layer was dried over sodium sulfate and concentrated. Flash chromatography (Deactivated Si_2_O, 20 % EtOAc in hexanes) afforded the benzimidazole **VIII** (65 mg, 62%) as a yellow foamy solid. ^1^H NMR (300 MHz, CDCl_3_) δ 10.71 (s, 1H), 7.85 – 7.72 (m, 1H), 7.59 – 7.39 (m, 4H), 7.32 (d, *J* = 7.3 Hz, 1H), 7.22 (dd, *J* = 7.6, 1.0 Hz, 2H), 4.96 (s, 1H), 3.72 (s, 3H), 2.65 (d, *J* = 34.1 Hz, 6H), 0.97 (s, 9H). ^13^C NMR (100 MHz, CDCl_3_) δ 172.8, 155.5, 151.6, 143.6, 137.4, 132.4, 129.8, 129.6, 128.8, 128.6, 128.4, 128.2, 76.1, 70.6, 52.4, 28.0, 23.6, 17.8.

**2-(*tert*-butoxy)-2-(6-(6-chloro-1H-benzo[d]imidazol-2-yl)-2,5-dimethyl-4-phenylpyridin-3-yl)acetic acid, (*-*)-KF116):**

A mixture of methyl esters **VIII** (44 mg, 0.092 mmol) in (1:3) MeOH/THF (1.0 mL) and 2*N* NaOH (0.15 mL) were stirred overnight at room temperature. The reaction mixture was then acidified with 1N HCl and diluted with brine and extracted with EtOAc (3 x 5 mL). The combined organic layers were washed with brine, dried over sodium sulfate, filtered and concentrated to give the **(*-*)-KF116**. ^1^H NMR (300 MHz, CDCl_3_) δ 10.68 (s, 1H), 7.87 – 7.68 (m, 1H), 7.59 – 7.42 (m, 4 H) 7.30 (m, 2H), 5.10 (s, 1H), 2.68 (s, 3H), 2.63 (s, 3H), 1.00(s, 9H). ^13^C NMR (100 MHz, CDCl_3_) δ 172.5, 154.5, 152.4, 144.3, 136.8, 131.5, 130.2, 130.1, 128.9, 128.7, 128.5, 128.3, 77.7, 70.31, 28.02, 23.38, 17.94. HRMS-ESI *m/z* (M+H)^+^ calcd for C_26_H_26_ClN_3_O_3_, 464.1741 found 464.1726. [a] HPLC trace >95%

**Table S1. Data collection and refinement statistics.**

HIV-1 IN-CCD(F185H):BI224436

| **PBD Accession: 6NUJ** |  |
| --- | --- |
|  |  |
| **Data Collection** |  |
| **Wavelength (Å)** | 1.542 |
| **Resolution range (Å)** | 24.37 - 2.099 (2.174 - 2.099) |
| **Space group** | P 31 2 1 |
| **Unit cell** |  |
| a, b, c (Å) | 72.211, 72.211, 66.079 |
| α, β, γ (°) | 90, 90, 120 |
| **Total reflections** | 178563 (15008) |
| **Unique reflections** | 12002 (1194) |
| **Multiplicity** | 14.9 (12.5) |
| **Completeness (%)** | 0.99 (1.00) |
| **Mean I/σ(I)** | 32.06 (9.04) |
| **Wilson B-factor** | 22.82 |
| **R-merge** | 0.05141 (0.2756) |
|  |  |
| **Refinement** |  |
| **Total reflections** | 11892 (1196) |
| **Rwork/Rfree** | 0.2190/0.2443 |
| **Number of atoms** |  |
| Protein | 1010 |
| Ligand | 53 |
| Water | 53 |
| **R.m.s Deviations** |  |
| Bonds (Å) | 0.002 |
| Angles (°) | 0.54 |
| **Ramachandran** |  |
| Favored (%) | 99 |
| Allowed (%) | 0.8 |
| Outlier (%) | 0 |
| **Rotamer outliers (%)** | 0 |
| **B-factors** |  |
| Protein | 29.08 |
| Ligand | 25.23 |
| Water | 33.63 |

*Numbers in parentheses represent the value in the highest resolution shell.
